# Biocompatible Snowman-like Dimer Nanoparticles for Improved Cellular Uptake in Intrahepatic Cholangiocarcinoma

**DOI:** 10.3390/pharmaceutics15082132

**Published:** 2023-08-14

**Authors:** Ruyin Chen, Xingqun Pu, Rongrong Liu, Xiaomeng Dai, Fangfu Ye, Chunxia Zhao, Peng Zhao, Jian Ruan, Dong Chen

**Affiliations:** 1 Department of Medical Oncology, The First Affiliated Hospital, Zhejiang University School of Medicine, Hangzhou 310003, China; 2 Wenzhou Institute, University of Chinese Academy of Sciences, Wenzhou 325001, China; 3 State Key Laboratory of Clean Energy Utilization, College of Energy Engineering, Zhejiang University, Hangzhou 310003, China; 4 Faculty of Engineering, Computer, and Mathematical Sciences, The University of Adelaide, Adelaide, SA 5005, Australia; 5 Zhejiang Key Laboratory of Smart Biomaterials, College of Chemical and Biological Engineering, Zhejiang University, Hangzhou 310027, China

**Keywords:** dimer nanoparticle, asymmetric nanoparticle, drug delivery, cellular uptake, cancer therapy

## Abstract

Intrahepatic cholangiocarcinoma (ICC) is one of the most aggressive types of human cancers. Although paclitaxel (PTX) was proven to exert potent anti-tumor effects against ICC, the delivery of PTX is still challenging due to its hydrophobic property. Nanoparticle (NP)-based carriers have been proven to be effective drug delivery vehicles. Among their physicochemical properties, the shape of NPs plays a crucial role in their performance of cellular internalization and thus anti-tumor efficacy of loaded drugs. In this study, dumbbell-like and snowman-like dimer NPs, composed of a polylactic acid (PLA) bulb and a shellac bulb, were designed and prepared as drug nanocarriers to enhance the efficiency of cellular uptake and anti-tumor performance. PLA/shellac dimer NPs prepared through rapid solvent exchange and controlled co-precipitation are biocompatible and their shape could flexibly be tuned by adjusting the concentration ratio of shellac to PLA. Drug-loaded snowman-like PLA/shellac dimer NPs with a sharp shape exhibit the highest cellular uptake and best cell-killing ability against cancer cells in an in vitro ICC model over traditional spherical NPs and dumbbell-like dimer NPs, as proven with the measurements of flow cytometry, fluorescent confocal microscopy, and the CCK8 assay. The underlying mechanism may be attributed to the lower surface energy required for the smaller bulbs of snowman-like PLA/shellac dimer NPs to make the initial contact with the cell membrane, which facilitates the subsequent penetration through the cellular membrane. Therefore, these dimer NPs provide a versatile platform to tune the shape of NPs and develop innovative drug nanocarriers that hold great promise to enhance cellular uptake and therapeutic efficacy.

## 1. Introduction

Intrahepatic cholangiocarcinoma (ICC) is one of the most aggressive types of human cancer [1,2]. Chemotherapy, such as paclitaxel (PTX), which is one of the well-established anti-cancer drugs to inhibit the proliferation of ICC [3,4], is the first-line therapy for advanced and recurrent ICC [5]. However, the performance of chemotherapy is strongly limited by the insufficiency in the development of therapeutic agents. Previous research have suggested that nanoparticle (NP)-mediated drug delivery systems, such as polymeric NPs, liposomes, micelles, dendrimers, nanogels, quantum dots, and iron oxide NPs [6,7,8], offer several advantages and overcome the disadvantages of traditional therapy through reducing the side effects, improving the efficacy, and prolonging the circulation time [9,10]. NPs are thus considered as a promising approach to enhance the potency of drugs in the treatment of ICC.

The shape of NPs has been identified as a pivotal parameter in influencing the cellular uptake, thereby affecting the cell proliferation and immunogenic response [11,12]. Traditional NPs are mainly spherical and the effects of size, surface charge, and surface functionality of spherical NPs on biological processes have been largely elucidated [13]. Recently, the cellular uptake of asymmetric NPs has attracted extensive attention, which appears to be a complex phenomenon that depends on multiple factors, such as contact surface area and particle adhesion energy [14,15]. In addition, the aspect ratio of asymmetric NPs has been reported to demonstrate impacts on cellular proliferation, migration, and apoptosis [11], and asymmetric NPs with a higher aspect ratio show an increased propensity to permeate through the cell membrane when compared with spherical NPs [11,16]. Therefore, the design and preparation of NPs with a desired shape are of great importance to improve the therapeutic potential of NP-based drug carriers for intracellular delivery.

Non-spherical NPs are generally fabricated using particle replication [17], microfluidics [18], film stretching, self-assembly [19], laser ablation [20], and high-pressure homogenization [21,22]. So far, non-spherical NPs, such as rod, tube, square, octahedron, star [23], cube, bowl [24], filament [25], cluster, disc, flower, needle, star, and spindle, which consist of polymer, carbon, silica, or metal, have been fabricated and reported [15]. However, biocompatible polymeric NPs with a tunable shape, such as dumbbell-like and snowman-like, are barely reported, and the performance of these NPs as drug delivery vehicles has not been fully explored as of yet.

Here, biocompatible dimer NPs with tunable morphology are designed as efficient drug nanocarriers, which demonstrate improved cellular internalization and enhanced anti-tumor efficacy. The proposed dimer NPs consist of a polylactic acid (PLA) bulb and a shellac bulb, which were prepared through rapid solvent exchange and controlled co-precipitation. Compared with other methods, co-precipitation is a one step, versatile, scalable, and green method to prepare biocompatible spherical NPs of different sizes [26]. In contrast, NPs with different morphology could be achieved via controlled co-precipitation, which combines co-precipitation with controlled phase separation [26]. Therefore, the shape of dimer NPs can be flexibly tuned through adjusting the concentration ratio of shellac to PLA, forming dumbbell-like and snowman-like structures. Hydrophobic drugs, such as paclitaxel (PTX), can be loaded in the dimer NPs during the co-precipitation process. The efficiency of PTX has been shown to be enhanced by the dimer particles significantly, and snowman-like NPs with a sharp shape exhibit the highest cellular uptake and best anti-tumor efficacy when compared with the traditional spherical NPs and dumbbell-like dimer NPs. The biocompatible dimer NPs proposed in this study provide a promising system to enhance the delivery efficiency and anti-tumor performance.

## 2. Materials and Methods

Shellac (wax free) was provided by Sigma-Aldrich. PLA, with a molecular weight of 3000 g/mol, was purchased from Jinan Daigang Biomaterial Co., Ltd. Paclitaxel, Jinan, China. Paclitaxel (PTX, 99% purity) was obtained from Xian Xianze Biotechnology Co., Ltd., Xi’an, China. Tetrahydrofuran (THF, purity 99.5%) and Tween 80 were supplied by Sinopharm Group Chemical Reagent Co., Ltd., Shanghai, China. The human intrahepatic biliary epithelial cells (HIBEC) and human cholangiocarcinoma cell line (KMCH1) were obtained from the Shanghai Institute for Biological Science, Shanghai, China. Dulbecco’s modified Eagle’s medium (DMEM) and fetal bovine serum were obtained from Thermo Fisher Scientific Co., Ltd., Waltham, MA USA. Penicillin–streptomycin was purchased from Shanghai Chuan Qiu Biotechnology Co., Ltd., Shanghai, China. Phosphate-buffered saline (PBS) was purchased from Shanghai Yuanye Biotechnology Co., Ltd., Shanghai, China. Cell counting kit-8 (CCK8) and 2-(4-amidinophenyl)-6-indolecarbamidine dihydrochloride (DAPI) were purchased from Beyotime Institute of Biotechnology, Shanghai, China. All other chemical reagents were of analytical grade and were used as received. Deionized (DI) water was used throughout the study.

### 2.1. Preparation of Drug-Loaded PLA/Shellac Dimer Nanoparticles (NPs)

The drug-loaded PLA/shellac dimer NPs (PLA/shellac-PTX NPs) were prepared using flash nanoprecipitation according to our previously reported method [27]. Briefly, shellac, PLA, and PTX were co-dissolved in THF. The above mixture and DI water containing 1 wt% Tween 80 were preheated in a 70 °C water bath. Next, 100 μL of the mixture was rapidly injected into 3 mL of DI water containing 1 wt% Tween 80 using a 1–200 μL gel-loading pipet tip and shaken immediately. The obtained PTX loaded PLA/shellac dimer NPs were then dialyzed in a dialysis bag with a molecular weight cut-off (MWCO) of 14,000 Da for 2 days to remove THF and allow the encapsulated drugs to precipitate, followed by centrifugation at 1000 r/min for 5 min to remove the unencapsulated drugs. The purified PLA/shellac-PTX NPs were then freeze dried for 48 h and reserved for further use. The Nile red (NR)-loaded PLA/shellac NPs were prepared through the same procedure, replacing PTX with NR.

The size and shape of PLA/shellac-PTX NPs can be tailored through adjusting the total concentration and the aspect ratio of PLA and shellac. To tune the size of the PLA/shellac-PTX NPs, the concentrations of both PLA and shellac varied from 1 mg/mL, 2 mg/mL, 3 mg/mL, 5 mg/mL, and 10 mg/mL to 15 mg/mL. The concentration of PTX was kept constant at 1 mg/mL. To tune the aspect ratio of PLA/shellac-PTX NPs, the total concentrations of the polymer (PLA and shellac) were kept constant at 10 mg/mL. The concentration of shellac varied from 1 mg/mL, 2 mg/mL, 3 mg/mL, 4 mg/mL, 5 mg/mL, 6 mg/mL, 7 mg/mL, and 8 mg/mL to 9 mg/mL. The concentration of PTX was also kept constant at 1 mg/mL.

### 2.2. Characterization of PLA/Shellac Dimer NPs

The size and zeta potential of dimer NPs were measured using dynamic light scattering (DLS, Nano ZS, Malvern, Great Malvern, UK) at a scattering angle of 90°. The data were obtained through averaging the three replicates. The morphologies of NPs in the dry state were observed using a scanning electron microscope (SEM, SU-70, Hitachi, Tokyo, Japan) operated at an acceleration voltage of 3 kV.

### 2.3. Drug Loading Capacity of PLA/Shellac Dimer NPs

To characterize the drug loading capacity of PLA/shellac dimer NPs, 0.1 mg of PLA/shellac-PTX NPs was dissolved in 100 μL of THF. After dissolving, 10 mL of acetonitrile was added, and the concentration of PTX in this solution was measured using a high-performance liquid column (HPLC, LC 1200, Agilent Technologies, Santa Clara, CA, USA). The mobile phase consisted of acetonitrile and DI water (50:50), and a reverse-phase C-18 column (C18, Agilent Technologies, Palo Alto, CA, USA) was also used (the flow rate of the mobile phase = 1.0 mL/min). A UV–Vis detector was then used to detect PTX at 227 nm. The drug loading capacity (LC) of PLA/shellac dimer NPs was calculated using Equation (1):

LC (%) = (weight of PTX in NPs/weight of NPs) × 100%.(1)

### 2.4. In Vitro Drug Release of Drug-Loaded PLA/Shellac Dimer NPs

The release behavior of drug-loaded NPs was evaluated using a dynamic dialysis method. NR, a hydrophobic fluorescent dye, was used as a model drug. In brief, 2 mL of PLA/shellac-NR NPs solution (1 mg/mL) was added in a dialysis bag (MWCO = 1.4 kDa) and then immersed in 88 mL of PBS buffer (pH 7.4) containing 1 wt% of Tween 80 in a 100 mL breaker. The temperature of the solution in breaker was maintained at 37 °C and gently stirred. At specific time intervals, 4 mL of medium outside the dialysis bag was removed and replaced with 4 mL of fresh PBS solution. The concentration of NR was measured using a UV–Vis spectrometer. The cumulative release (CR) was calculated using Equation (2):

(2) $$CR(\%)=\frac{V_{0}C_{i}+V\sum_{1}^{i-1}C_{i}}{M}\times 100\%$$

where V_0_ is the total volume of the release medium (90 mL), *C_i_* is the drug concentration of the medium taken out at time *i* (μg/mL), V is the volume of medium taken out at each time (4 mL), and M is the total mass of drug in the NPs (μg).

The drug release data were fitted with a first-order model (Equation (3)) to determine the drug release mechanism of the drug-loaded PLA/shellac NPs:

(3) $$\ln(1-\frac{M_{t}}{M_{\infty }})=-kt$$

where *M_t_*/*M*_∞_ is the fraction of drug release at time *t*, and *k* is the diffusion constant.

### 2.5. Biocompatibility of PLA/Shellac Dimer NPs

HIBECs were used to test the biocompatibility of PLA/shellac dimer NPs using CCK8 assays. Cells were cultured in DMEM liquid medium supplemented with 10% fetal bovine serum and 1% penicillin–streptomycin at 37 °C in incubators with 5% carbon dioxide. HIBECs were seeded onto a 96-well plate at 1000 cells per well with 100 μL culture medium and incubated for 24 h. The culture medium was then replaced with fresh liquid medium containing PLA/shellac dimer NPs (shellac: PLA = 5:5, S5) at concentrations of 0, 0.025, 0.05, 0.1, and 0.2 mg/mL. After 24 h and 48 h of co-culture, the culture medium was removed and replaced with 100 μL of fresh culture medium containing 10 μL of CCK8 solution. A microplate reader (Synergy Neo2, Biotek, Winooski, VT, USA) was then used to measure the absorbance at 450 nm after incubation for 2 h. Cell viability was calculated through Equation (4):

(4) $$Cell\;Viability\left(\%\right)=\frac{Mean\;OD_{sample}-Mean\;OD_{NP}}{Mean\;OD_{NC}-Mean\;OD_{blank}}\times 100\%$$

where *Mean OD_sample_*, *Mean OD_NP_*, *Mean OD_NC_*, and *Mean OD_blank_* refer to the mean absorbances of PLA/shellac NPs-treated cells, culture medium containing only PLA/shellac dimer NPs, untreated cells, and blank culture medium, respectively.

### 2.6. Cellular Uptake of NR-Loaded PLA/Shellac Dimer NPs

NR, a fluorescent dye, was loaded in the PLA/shellac-NR NPs to investigate the cellular uptake of the dimer NPs. KMCH1 cells were seeded onto 12-well plates and incubated for 24 h and were then co-cultured with different shapes of PLA/shellac-NR at a concentration of 5 μg/mL. The same volume of blank culture medium served as a control group. After incubation for 24 h, the cells were digested and prepared into single-cell suspensions. Flow cytometry was performed to measure the quantitative cellular uptake efficiency of PLA-shellac-NR NPs by KMCH1 cells (CytoFlex LX, Beckman Coulter, Pasadena, CA, USA).

Fluorescent confocal microscopy was used to further confirm the cellular uptake of PLA/shellac-NR NPs. Briefly, KMCH1 cells were seeded onto confocal dishes and incubated for 24 h. KMCH1 cells were co-cultured with the same concentration of PLA/shellac-NR NPs (5 mg/mL) but with different shapes. The same volume of blank culture medium served as the control group. The cells were then washed three times using PBS and then fixed with 4% paraformaldehyde for 15 min. The nucleus was stained with DAPI (2 ug/mL) for 15 min. After washing with PBS for three times, the cells were imaged using fluorescent confocal microscopy (LSM 900 Airyscan2, Zeiss, Jena, Germany)

### 2.7. Cytotoxicity of Paclitaxel (PTX)-Loaded PLA/Shellac Dimer NPs

KMCH1 cells were seeded at 1000 cells per well in the 96-well plate with 100 μL of culture medium and incubated for 24 h. After being co-cultured with the liquid medium containing free PTX and PTX-loaded NPs at the same PTX concentration (0.02 μg/mL) for 48 and 72 h, CCK8 assays were performed to detect the cell viability of KMCH1 cells, following the methods described above. A microplate reader (Synergy Neo2, Biotek, Winooski, VT, USA) was used to measure the absorbance at 450 nm following incubation for 2 h.

### 2.8. Statistical Analysis

Statistical analysis was conducted using GraphPad Prism 9.0.0. The data are presented as mean ± standard error of the mean (SEM). Statistical analysis was performed using the one-way ANOVA. Differences were considered as statistically significant if *p* < 0.05 (ns denotes not significant, *, **, ***, and **** denotes *p* < 0.05, *p* < 0.01, *p* < 0.001, and *p* < 0.0001, respectively).

## 3. Results

### 3.1. Preparation and Characterization of PTX-Loaded PLA/Shellac Dimer NPs

**Figure 1:**
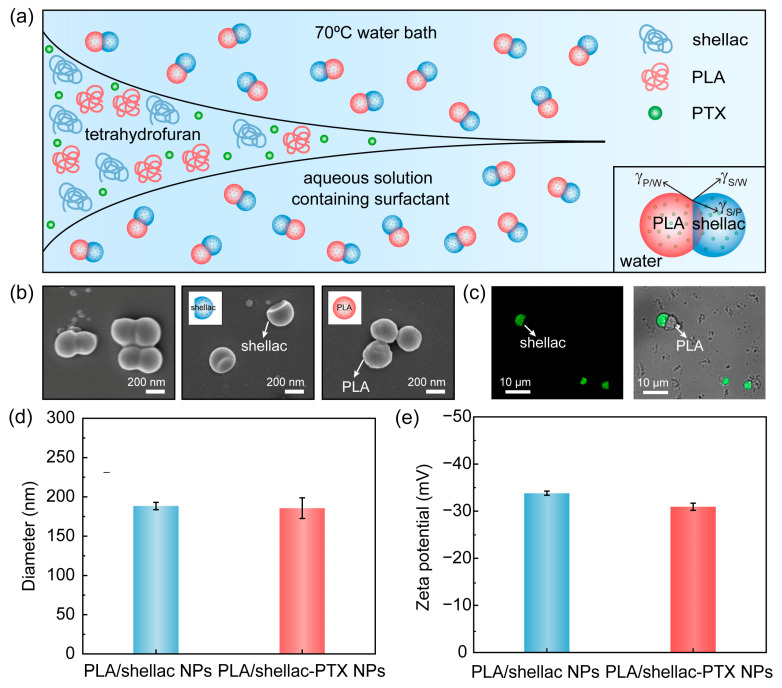
Design and preparation of PLA/shellac dimer NPs for drug delivery. (**a**) Schematic illustration of the preparation of PLA/shellac-PTX dimer NPs via rapid mixing and controlled co-precipitation. (**b**) SEM images of PLA/shellac NPs (**left**), shellac bulbs after removing PLA bulbs by ethyl acetate (**middle**), and PLA bulbs after removing shellac bulbs using alkaline solution (**right**). (**c**) Fluorescent (**left**) and overlay (**right**) images of PLA/shellac dimer NPs. (**d**) Particle size distribution measured using dynamic light scattering, and (**e**) Zeta potential of PLA/shellac NPs and PLA/shellac-PTX NPs. Where not specified, PLA/shellac dimer NPs were prepared using 5 mg/mL PLA and 5 mg/mL shellac in tetrahydrofuran.

PLA, a type of polymeric material with excellent biocompatibility, biodegradability, and process ability [28], and shellac, which is a biocompatible and biodegradable polymer that consists of polyesters and single esters, are both FDA-approved, as shown in Figure S1 [29]. PTX-loaded PLA/shellac dimer NPs (PLA/shellac-PTX dimer NPs) were prepared through rapid solvent exchange and controlled co-precipitation, as shown in Figure 1a. PLA, shellac, and PTX were co-dissolved in tetrahydrofuran (THF) and quickly injected into a reservoir of DI water containing the 1 wt% non-ionic surfactant Tween 80. Upon rapid solvent exchange of THF by water, PLA, shellac, and PTX co-precipitated and phase-separated to form PLA/shellac-PTX dimer NPs. Generally, the co-precipitation of two binary polymers could form four different morphologies, including occluded, core shell, dimer, and heteroaggregate [13,30,31,32]. The presence of the non-ionic surfactant in the water phase is able to tune the PLA/water (P/W) and shellac/water (S/W) interfacial tensions to favor g_P/W_~g_S/W_ > g_P/S_ and thus form a dimer structure [26,33]. PLA/shellac-PTX dimer NPs that were prepared using the same concentration of PLA and shellac consist of two distinct bulbs, one PLA bulb and one shellac bulb, and show a dumbbell-like shape, as shown in Figure 1b. The crescent moon-like shellac bulb was confirmed by dissolving PLA with ethyl acetate, while the sphere-like PLA bulb was confirmed by dissolving shellac with alkaline solution. The dimer structure was further confirmed with florescence imaging, in which the shellac bulb with fluorescent property showed green fluorescence while the PLA bulb appeared dark, as shown in Figure 1c. This further confirmed that the PLA/shellac-PTX NPs were composed of the PLA and shellac bulbs. The average sizes of the PLA/shellac NPs and PLA/shellac-PTX NPs in DI water that were measured using dynamic light scattering (DLS) were 188.5 nm (PDI = 0.32) and 185.7 nm (PDI = 0.36), respectively, as shown in Figure 1d and Figure S2. In addition, PLA/shellac and PLA/shellac-PTX NPs were found to have a similar Zeta potential of about *−*30 mV, which was attributed to the partial ionization of the carboxylic groups of shellac, as shown in Figure 1e. Additionally, no significant change in size was observed after incubating PLA/shellac NPs in biologically relevant media when the pH varied from 7.4 to 6.5 for five days, indicating that the PLA/shellac NPs demonstrate excellent stability under a physiological environment, as shown in Figure S3.

**Figure 2:**
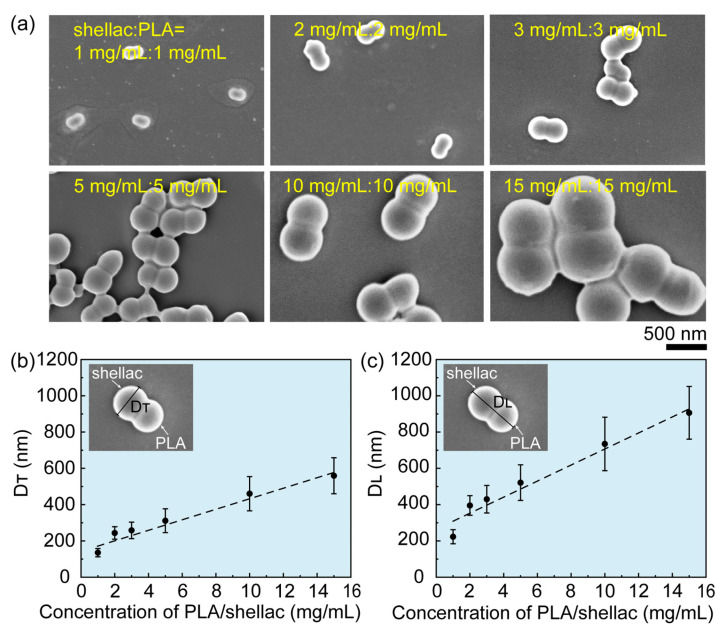
Flexible tuning of the size of PLA/shellac-PTX dimer NPs. (**a**) SEM images of PLA/shellac-PTX dimer NPs prepared using the same concentrations of shellac and PLA. The concentration of shellac and PLA was increased from 1 mg/mL to 15 mg/mL. (**b**) Transversal length, D_T_, and (**c**) longitudinal length, D_L_, of PLA/shellac-PTX dimer NPs as a function of shellac concentration. The concentration of PLA was the same as for shellac.

The size of the PLA/shellac-PTX NPs can flexibly be tuned by changing the concentration of PLA and shellac, as shown in Figure 2a. Overall, the size of the PLA/shellac-PTX NPs increased linearly as the concentration of PLA and shellac also increased. Specifically, when the concentration of PLA and shellac increased from 1 mg/mL to 15 mg/mL, the transversal length, D_T_, of dumbbell-like PLA/shellac-PTX dimer NPs increased linearly from 135 nm to 558 nm, while the longitudinal length, D_L_, also increased linearly from 220 nm to 900 nm, as shown in Figure 2b,c, respectively. The linear dependence of particle size on polymer concentration was attributed to the rapid solvent exchange in that solvent exchange was completed before polymer aggregation, thus leading to concentration-limited particle formation [31].

**Figure 3:**
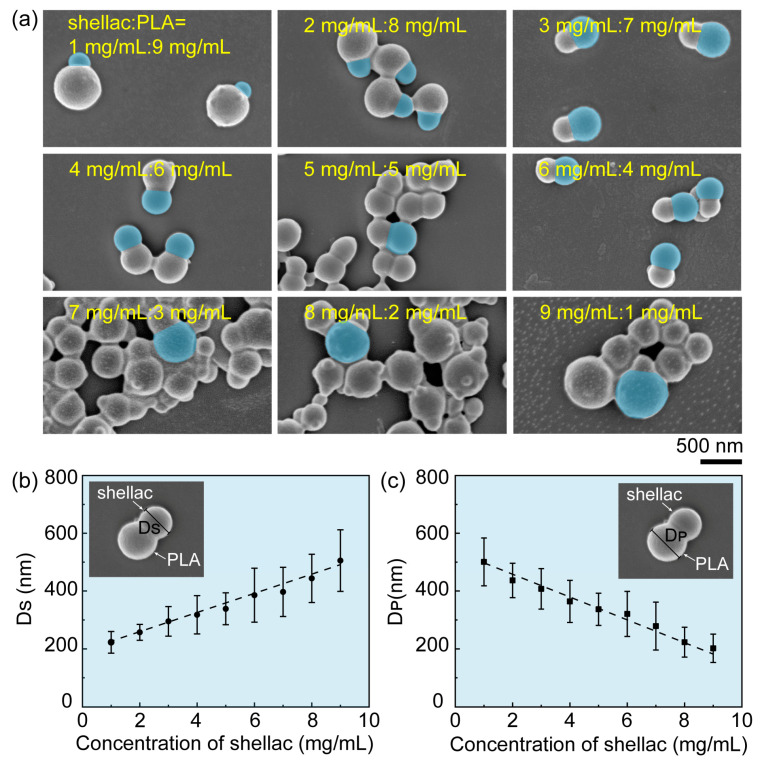
Flexible tuning of the shape of PLA/shellac-PTX dimer NPs. (**a**) SEM images of PLA/shellac-PTX dimer NPs prepared using different concentrations of shellac and PLA. The concentration of shellac was increased from 1 mg/mL to 9 mg/mL while the concentration of PLA was decreased from 9 mg/mL to 1 mg/mL, respectively. Their total concentration was kept constant at 10 mg/mL. (**b**) Diameter of shellac bulbs, D_S_, and (**c**) diameter of PLA bulbs, D_P_, as a function of shellac concentration. The total concentration of PLA plus shellac was kept constant at 10 mg/mL.

In addition to the size, the shape of PLA/shellac-PTX NPs could also be flexibly tailored by changing the concentration ratio of shellac to PLA, as shown in Figure 3a. When the total concentration of shellac plus PLA was kept constant at 10 mg/mL and the concentration ratio of shellac to PLA was changed from 1:9 to 9:1, the particle shape changed from snowman-like to dumbbell-like and then back to snowman-like. Specifically, the diameter of shellac bulbs, D_S_, increased linearly from 220 nm to 505 nm, while the diameter of PLA bulbs, D_P_, decreased linearly from 500 nm to 200 nm, when the shellac-to-PLA concentration ratio was changed from 1:9 to 9:1, as shown in Figure 3b,c, respectively.

**Figure 4:**
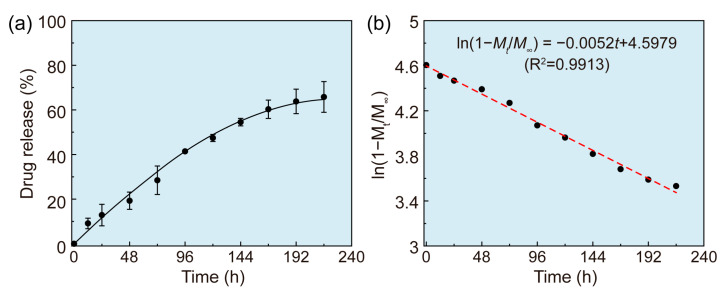
Release profile of PLA/shellac-NR dimer NPs. (**a**) Release profile of Nile red (NR) loaded in PLA/shellac dimer NPs. NR was chosen as a model drug to facilitate measurements. (**b**) Fitting of the release profile of PLA/shellac-NR dimer NPs using a first-order kinetic model.

During the particle preparation process, PTX was successfully loaded in the PLA/shellac-PTX dimer NPs due to the same hydrophobic nature of PTX, PLA, and shellac. The loading capability of PTX in the dimer NPs was about 4.32%. NR was used as a model drug to measure the release profile of the PLA/shellac NPs. The drug release profile of NR from the dimer NPs was investigated and shown in Figure 4a. After 24 h, about 13% of NR was released, and the total amount of released drug reached up to 68% in 10 days, showing a typical profile of sustained release. To determine the release kinetics of the PLA/shellac-NR dimer NPs, the release profile was fitted with a first-order kinetic model, as shown in Figure 4b. The linear correlation coefficient (R^2^) was 0.9913, which suggests that the release behavior of the PLA/shellac-NR dimer NPs is in accordance with the first-order release kinetics, and that the drug release mechanism is expected to be controlled through Fickian diffusion [34]. Additionally, the PLA/shellac NPs demonstrated great stability in 1 wt% Tween 80 following a five-day incubation period, suggesting that 1 wt% Tween 80 does not disturb the stability of NPs in the release test, as shown in Figure S4.

### 3.2. Biocompatibility and Cellular Uptake of PLA/Shellac Dimer NPs

**Figure 5:**
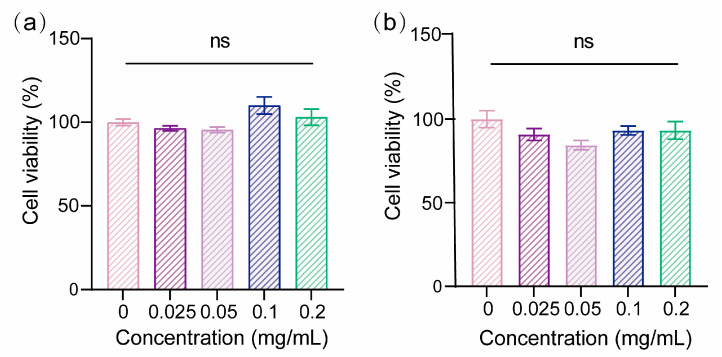
Biocompatibility of the PLA/shellac dimer NPs tested using HIBECs. Cell viability of HIBECs after (**a**) 24 h and (**b**) 48 h treated with blank PLA/shellac dimer NPs at concentrations of 0, 0.025, 0.05, 0.1, and 0.2 mg/mL. Blank PLA/shellac dimer NPs were prepared using 5 mg/mL PLA and 5 mg/mL shellac. Where not specified, data are represented as mean ± SEM (*n* = 3), and ns denotes not significant.

Both PLA and shellac are FDA-approved materials, and PLA/shellac dimer NPs are expected to be biocompatible. To further assess the biocompatibility of PLA/shellac dimer NPs, HIBECs, obtained from a human intrahepatic biliary epithelial cell line, were co-cultured with blank PLA/shellac dimer NPs at concentrations of 0, 0.025, 0.05, 0.1, and 0.2 mg/mL and their cell viability after 24 h and 48 h are shown in Figure 5a,b, respectively. No significant differences were observed, even when the concentration of blank PLA/shellac dimer NPs was up to 0.2 mg/mL, suggesting a good biocompatibility of the dimer NPs.

**Figure 6:**
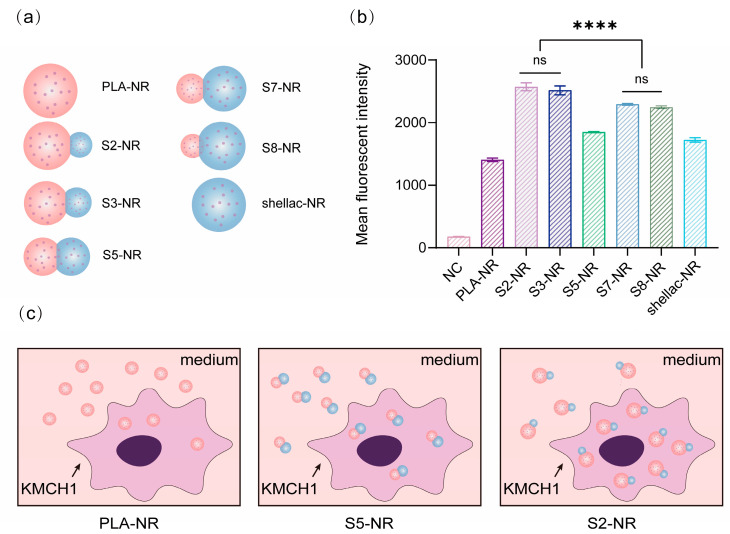
Cellular uptake of NR-loaded PLA/shellac dimer NPs measured using cell flow cytometry. (**a**) Schematic illustration of PLA/shellac-NR dimer NPs with different shapes. PLA-NR: spherical PLA NPs loaded with NR; S2-NR: snowman-like dimer NPs prepared with shellac:PLA = 2:8; S3-NR: snowman-like dimer NPs prepared with shellac:PLA = 3:7; S5-NR: dumbbell-like dimer NPs prepared with shellac:PLA = 5:5; S7-NR: snowman-like dimer NPs prepared with shellac:PLA = 7:3; S8-NR: snowman-like dimer NPs prepared with shellac:PLA = 8:2; and shellac-NR: spherical shellac NPs loaded with NR. (**b**) Mean fluorescent intensity of KMCH1 cells after 24 h of co-culture with the same concentration of NPs (5 mg/mL) but different shapes. Cells treated with the blank culture medium served as the negative control (NC). (**c**) Schematic illustration of the cellular internalization of PLA/shellac-NR dimer NPs with different shapes in KMCH1 cells. Where not specified, ns denotes not significant, **** denotes *p* < 0.0001.

To investigate the cellular uptake of the dimer NPs, Nile red (NR), a fluorescent dye, was loaded in the PLA/shellac-NR NPs to supplement the NPs with a fluorescent probe. KMCH1 cells were co-cultured with different shapes of NPs at the same concentration of 5 mg/mL, including spherical PLA NPs (PLA-NR), snowman-like dimer NPs prepared with shellac:PLA = 2:8 (S2-NR), shellac:PLA = 3:7 (S3-NR), shellac:PLA = 7:3 (S7-NR) and shellac:PLA = 8:2 (S8-NR), dumbbell-like dimer NPs prepared with shellac:PLA = 5:5 (S5-NR), and spherical shellac NPs (shellac-NR), whose shapes have been schematically modeled in Figure 6a. To minimize the impact of particle size on cellular uptake, different NPs were prepared using the same total concentration of C_PLA_ plus C_shellac_. After 24 h of co-culture, flow cytometry assays were performed to characterize the cellular uptake of NR-loaded dimer NPs with different shapes by KMCH1 cells, as shown in Figure 6b. The mean fluorescent intensity (MFI) of the KMCH1 cells treated with different shapes of NPs suggested that snowman-like dimer NPs (S2-NR, S3-NR, S7-NR, and S8-NR) show a better performance of cellular uptake than dumbbell-like dimer NPs (S5-NR), while dumbbell-like dimer NPs (S5-NR) were deemed to be superior to spherical NPs (PLA-NR and shellac-NR), as shown in Figure 6c. These results are consistent with previous simulations which revealed that NPs with a sharp shape, such as snowman-like NPs, demonstrate a better performance of cell internalization [11,16]. Although the underlying mechanism needs to be further clarified, it may be attributed to the lower surface energy required for smaller bulbs to make the initial contact with the cell membrane, which facilitates the subsequent penetration of whole dimer NPs through the cell membrane, thus leading to better cellular uptake [14,35,36,37]. Interestingly, snowman-like dimer NPs with a smaller shellac bulb (S2-NR and S3-NR) showed a better performance than NPs with a similar shape but a larger shellac bulb (S7-NR and S8-NR); this was deemed to be due to the shellac bulb and PLA bulb encompassing different surface properties. The shellac bulb is rich with carboxylic groups and negatively charged. However, the cell membrane is also negatively charged, thus disfavoring the large shellac bulb.

**Figure 7:**
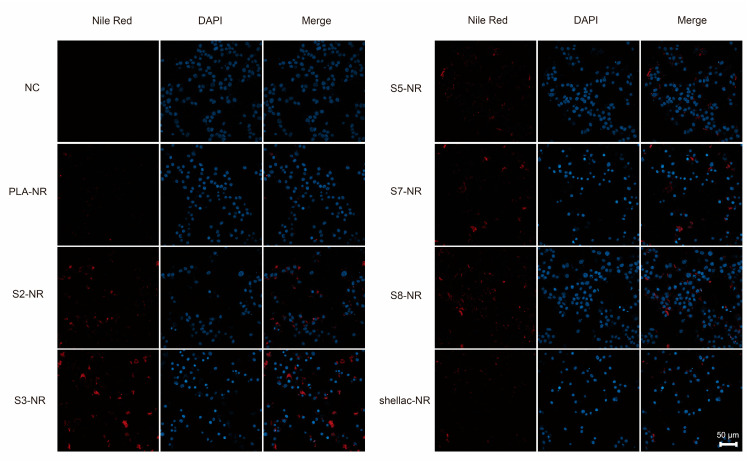
Cellular uptake of NR-loaded PLA/shellac dimer NPs characterized using fluorescent confocal microscopy. Fluorescent confocal microscope images of KMCH1 cells after 24 h of incubation with the same concentration of NPs (5 mg/mL) but different shapes. The red signal represents NPs loaded with NR, while the blue signal represents nuclei stained with DAPI. The scale bar is 50 μm.

To directly visualize the cellular uptake of NR-loaded NPs by KMCH1 cells, KMCH1 cells after 24 h of incubation with different shapes of NPs were imaged using fluorescent confocal microscopy, where the red signal represents NPs loaded with NR and the blue signal represents nuclei stained with DAPI, as shown in Figure 7. The NPs were observed to localize within the cytoplasm and snowman-like dimer NPs showed the best performance of cellular uptake over the dumbbell-like and spherical NPs, which is consistent with the results measured using flow cytometry.

### 3.3. Anti-Tumor Effects of PTX-Loaded PLA/Shellac Dimer NPs

The above results suggest that PLA/shellac dimer NPs are biocompatible and that they are excellent nanocarriers for drug delivery since the performance of cellular uptake could be improved through tuning the shape of NPs. Therefore, drug-loaded snowman-like dimer NPs are expected to show increased cellular internalization and enhanced anti-tumor efficacy for cancer therapy. To test this speculation, PTX, which exerts anti-tumor effects against intrahepatic cholangiocarcinoma (ICC) by impeding the epithelial–mesenchymal transition, disrupting the desmoplastic stroma, and inhibiting the tumor cell proliferation, invasiveness, and hematogenous metastasis [38,39], was chosen as a model drug and loaded in PLA/shellac dimer NPs upon their co-precipitation.

**Figure 8:**
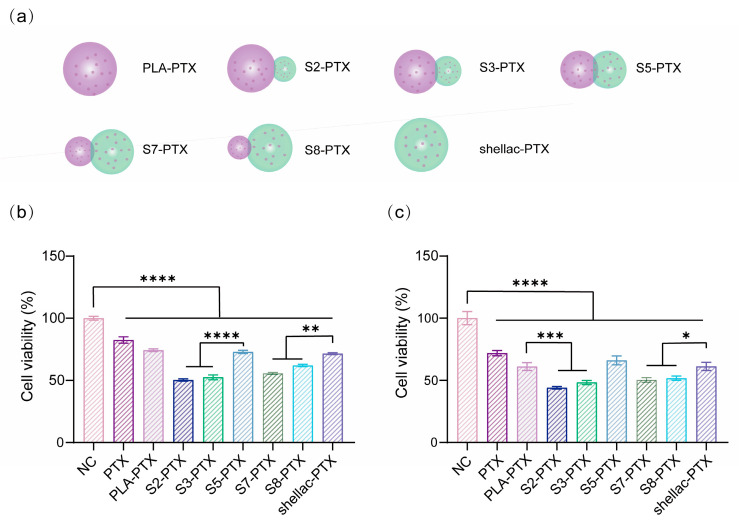
In vitro anti-tumor performance of PTX-loaded PLA/shellac dimer NPs. (**a**) Schematic illustration of PLA/shellac-PTX dimer NPs with different shapes. (**b**) Cell viability of KMCH1 cells after 48 h of incubation with PLA/shellac-PTX NPs with the same concentration of PTX (0.02 μg/mL) but different shapes. (**c**) Cell viability of KMCH1 cells after 72 h of treatment with PLA/shellac-PTX NPs with the same concentration of PTX (0.02 μg/mL) but different shapes. Cells treated with PBS served as the negative control. Where not specified, *, **, *** and **** denote *p* < 0.05, *p* < 0.01, *p* < 0.001 and *p* < 0.0001, respectively.

The delivery performance and anti-tumor efficacy of PLA/shellac-PTX dimer NPs with different shapes, including free PTX, PTX-loaded PLA (PLA-PTX), PTX-loaded shellac (shellac-PTX), and PTX-loaded PLA/shellac dimer NPs with a shellac-to-PLA ratio of 2:8 (S2-PTX), 3:7 (S3-PTX), 5:5 (S5-PTX), 7:3 (S7-PTX), and 8:2 (S8-PTX), as modeled in Figure 8a, were explored via the CCK8 assay. The cell viabilities of KMCH1 cells co-cultured with PLA/shellac-PTX NPs with the same concentration of PTX (0.02 μg/mL) but with different shapes at 48 h and 72 h were determined and displayed in Figure 8b,c respectively. The anti-tumor efficacy of PTX-loaded dimer NPs was found to be correlated with the cellular uptake and cytoplasmic accumulation of NPs, and that snowman-like PLA/shellac-PTX dimer NPs (S2-PTX, S3-PTX, S7-PTX, and S8-PTX) inhibited KMCH1 cells significantly and displayed the best anti-tumor performance when compared with dumbbell-like dimer NPs (S5-PTX) and spherical NPs (PLA-PTX and shellac-PTX). These results are consistent with those obtained using flow cytometry and fluorescent confocal microscopy, and further prove that snowman-like dimer NPs are effective delivery vehicles for cancer therapy, which have greatly enhanced the cell-killing ability of PTX to ICC cells.

## 4. Conclusions

PLA/shellac dimer NPs composed of a PLA bulb and a shellac bulb have been designed and prepared via rapid solvent exchange and controlled co-precipitation. The size and shape of dimer NPs are able to be flexibly tuned, forming dumbbell-like and snowman-like structures. PLA/shellac dimer NPs are biocompatible and serve as good drug nanocarriers to enhance the efficiency of cellular internalization and anti-tumor efficacy. Of note, drug-loaded snowman-like PLA/shellac dimer NPs with a sharp shape showed the highest cellular uptake and best cell-killing ability against cancer cells over dumbbell-like dimer NPs and spherical NPs, as proven with the measurements of flow cytometry, fluorescent confocal microscopy, and the CCK8 assay. These results suggest that PLA/shellac dimer particles hold great potential as drug delivery vehicles for therapeutic applications, and further studies will shine light on the design of novel drug nanocarriers for enhanced cellular uptake and improved anti-tumor efficacy.

## Data Availability

The data presented in this study are available on request from the corresponding author.

## Supplementary Materials

The following supporting information can be downloaded at: https://www.mdpi.com/article/10.3390/pharmaceutics15082132/s1, Figure S1: Chemical structures of (a) shellac and (b) PLA. Both PLA and shellac are FDA-approved materials; Figure S2: Particle size distribution of PLA/shellac NPs and PLA/shellac-PTX NPs measured by dynamic light scattering. No significant change in size measured by dynamic light scattering is observed after incubating PLA/shellac NPs in PBS with pH varied from 7.4 to 6.5 and 1 wt% Tween 80 for 5 days; Figure S3: Stability of PLA/shellac dimer NPs in physiological and cancerous conditions. No significant change in size measured by dynamic light scattering is observed after incubating PLA/shellac NPs in PBS with pH varied from 7.4 to 6.5 for 5 days; Figure S4: Stability of PLA/shellac dimer NPs in 1 wt% Tween 80. No significant change in size measured by dynamic light scattering is observed after incubating PLA/shellac NPs in 1 wt% Tween 80 for 5 days.
