# Supplementary material for: Biocompatible Snowman-like Dimer Nanoparticles for Improved Cellular Uptake in Intrahepatic Cholangiocarcinoma
Source: Pharmaceutics. 2023 Aug 14;15(8):2132. doi: 10.3390/pharmaceutics15082132 (PMC10459898; doi:10.3390/pharmaceutics15082132)
Supplement: Supplementary file 1 [file pharmaceutics-15-02132-s001.zip › pharmaceutics-2479634-supplementary.pdf]

Support information

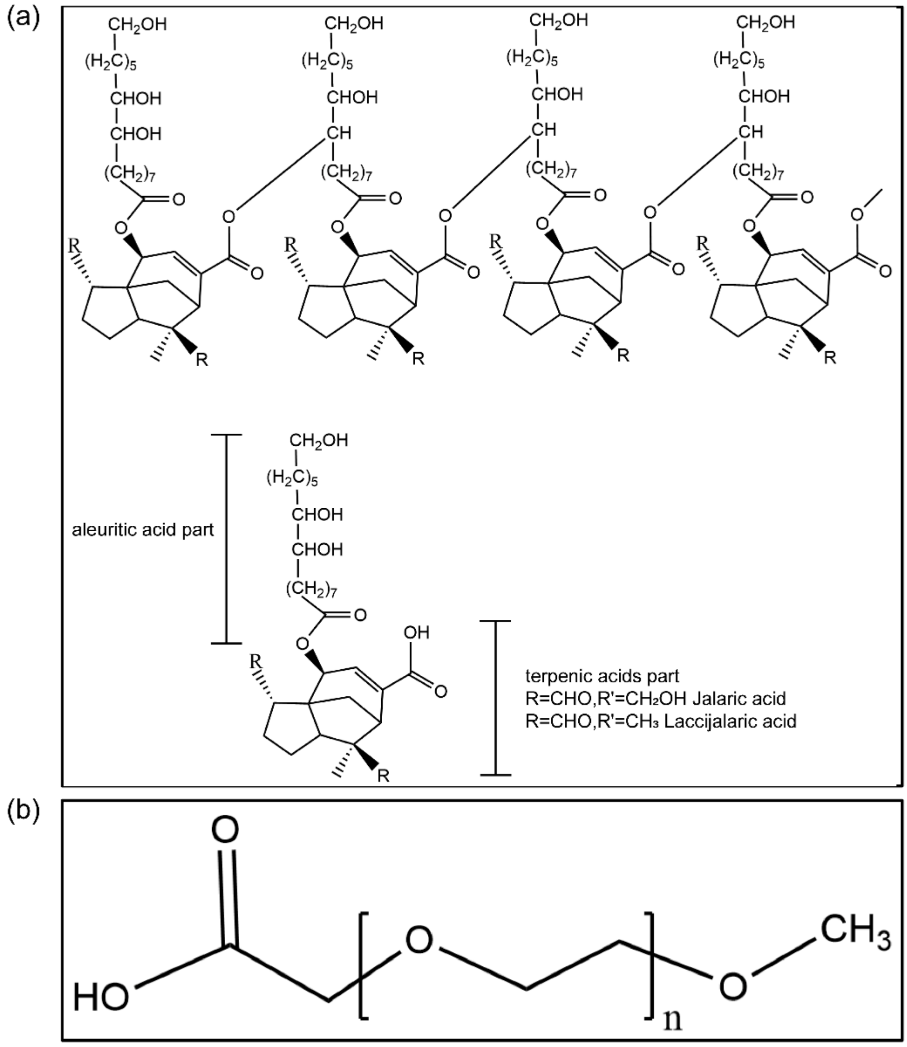

Figure S1. Chemical structures of (a) shellac and (b) PLA. Both PLA and shellac are FDA-approved materials.

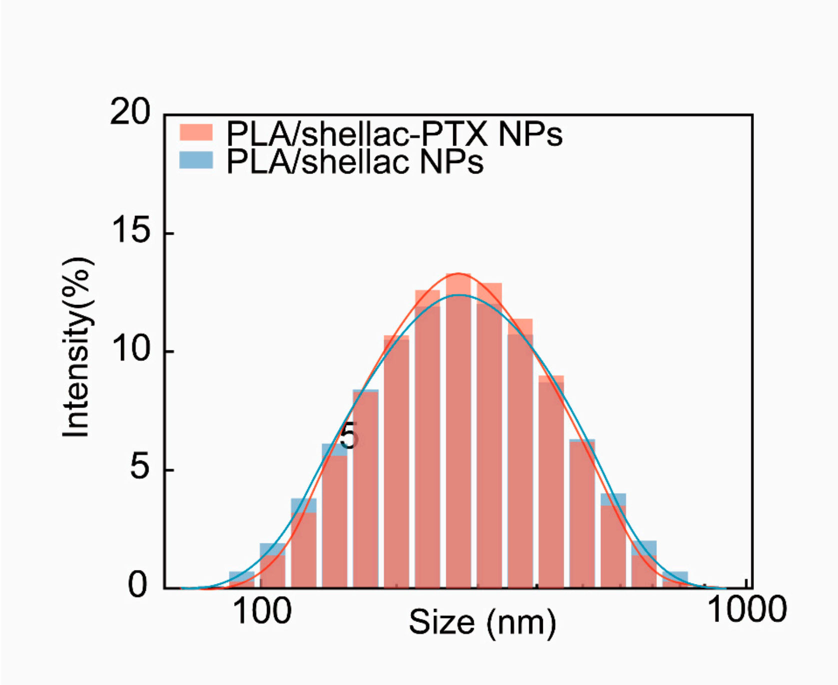

Figure S2. Particle size distribution of PLA/shellac NPs and PLA/shellac-PTX NPs measured by dynamic light scattering. No significant change in size measured by dynamic light scattering is observed after incubating PLA/shellac NPs in PBS with pH varied from 7.4 to 6.5 and 1 wt% Tween 80 for 5 days.

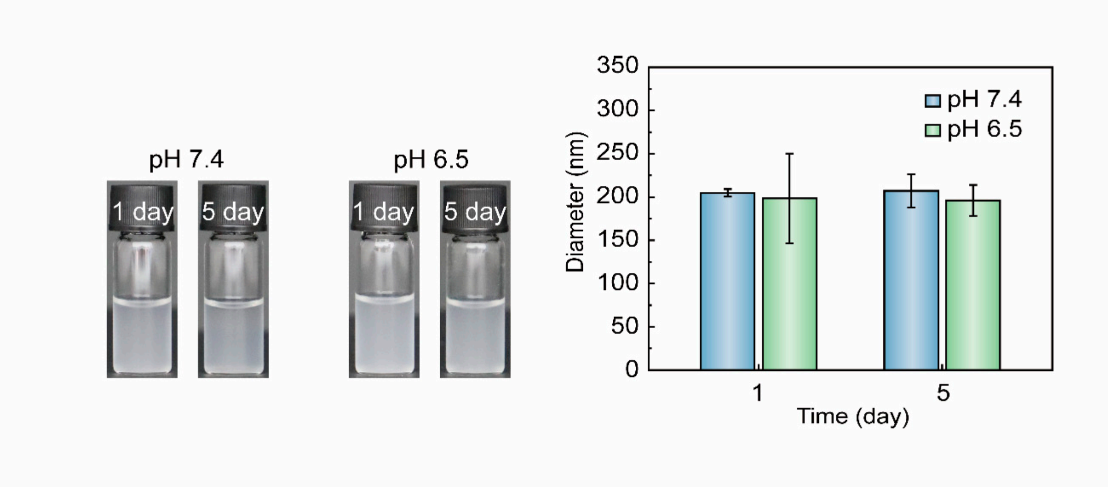

Figure S3. Stability of PLA/shellac dimer NPs in physiological and cancerous conditions. No significant change in size measured by dynamic light scattering is observed after incubating PLA/shellac NPs in PBS with pH varied from 7.4 to 6.5 for 5 days.

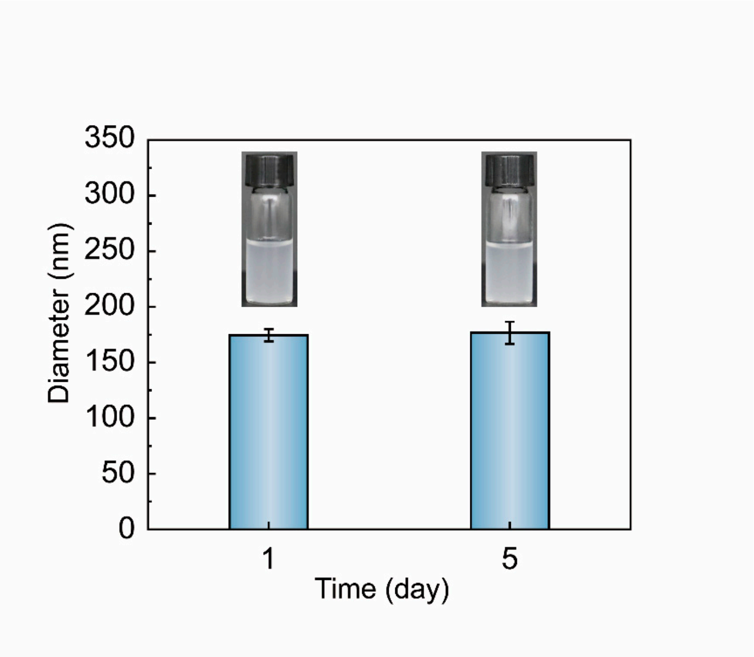

Figure S4. Stability of PLA/shellac dimer NPs in 1 wt% Tween 80. No significant change in size measured by dynamic light scattering is observed after incubating PLA/shellac NPs in 1 wt% Tween 80 for 5 days.
